# Bronchial artery embolization using small particles is safe and effective: a single center 12-year experience

**DOI:** 10.1007/s00330-024-10836-y

**Published:** 2024-06-11

**Authors:** Frances Sheehan, Alison Graham, N. Paul Tait, Philip Ind, Ali Alsafi, James E. Jackson

**Affiliations:** 1 Imaging Department, Imperial College Healthcare NHS Trust, London, UK; 2 Department of Respiratory Medicine, Imperial College Healthcare NHS Trust, London, UK; 3 Department of Surgery and Cancer, Imperial College London, London, UK

**Keywords:** Bronchial arteries, Embolization (therapeutic), Hemoptysis, Polyvinyl alcohol

## Abstract

**Background:**

Bronchial artery embolization (BAE) using particles is an established treatment for hemoptysis. The use of polyvinyl alcohol (PVA) with a particle size of 300 µm or larger is thought to reduce the risk of non-target embolization but may result in more proximal vessel occlusion than is ideal, resulting in a high rate of early recurrent hemorrhage.

**Objective:**

This study evaluates the safety and efficacy of BAE using PVA particles with a size of less than 300 µm.

**Methods:**

All patients who underwent BAE between 2010 and 2022 at a tertiary center were included. Demographic data, etiology and volume of hemoptysis, technical and clinical success, procedure-related complications, and follow-up information were collected from patients’ electronic records. 150–250 µm PVA particles were used to commence embolization in all patients with the subsequent use of larger-sized particles in some individuals. The Kaplan–Meier method was used to estimate recurrence and survival rates.

**Results:**

One hundred forty-four patients underwent 189 embolization procedures between 2010 and 2022 and were followed up for a median of 35 months [IQR 19–89]. 150 µm to 250 µm PVA particles were used as the sole embolic agent in 137 cases. Hemoptysis recurred within 30 days in 7%. The median time to repeat intervention was 144 days [IQR 42–441]. Seventeen out of 144 patients had a pulmonary artery branch pseudoaneurysm. The rate of major complications was 1% with no instances of stroke or spinal artery ischemia. Thirty-day mortality was 2% (4/189).

**Conclusion:**

BAE using 150–250 µm PVA particles is safe and effective with few complications and low rates of early hemoptysis recurrence.

**Clinical relevance statement:**

BAE using small particles is likely to improve outcomes, particularly the rate of early recurrence, in patients with hemoptysis, without an increase in procedural complications.

**Key Points:**

- *BAE is a safe and effective treatment for patients with hemoptysis*.
- *Using small PVA particles in BAE has few complications and low rates of early recurrence*.
- *Pulmonary artery pseudoaneurysms should be actively sought in those with hemoptysis undergoing BAE*.

## Introduction

Bronchial artery embolization (BAE) is widely used in the treatment of significant hemoptysis. Its use is well-established as a primary method of bleeding control, in secondary prevention, and as a bridge to surgery. It can be repeated in cases of recurrent hemoptysis, and it generally preserves pulmonary function [1, 2]—an important factor in patients who often have limited pulmonary reserve, and for whom surgery has a high mortality rate.

**Fig. 1:**
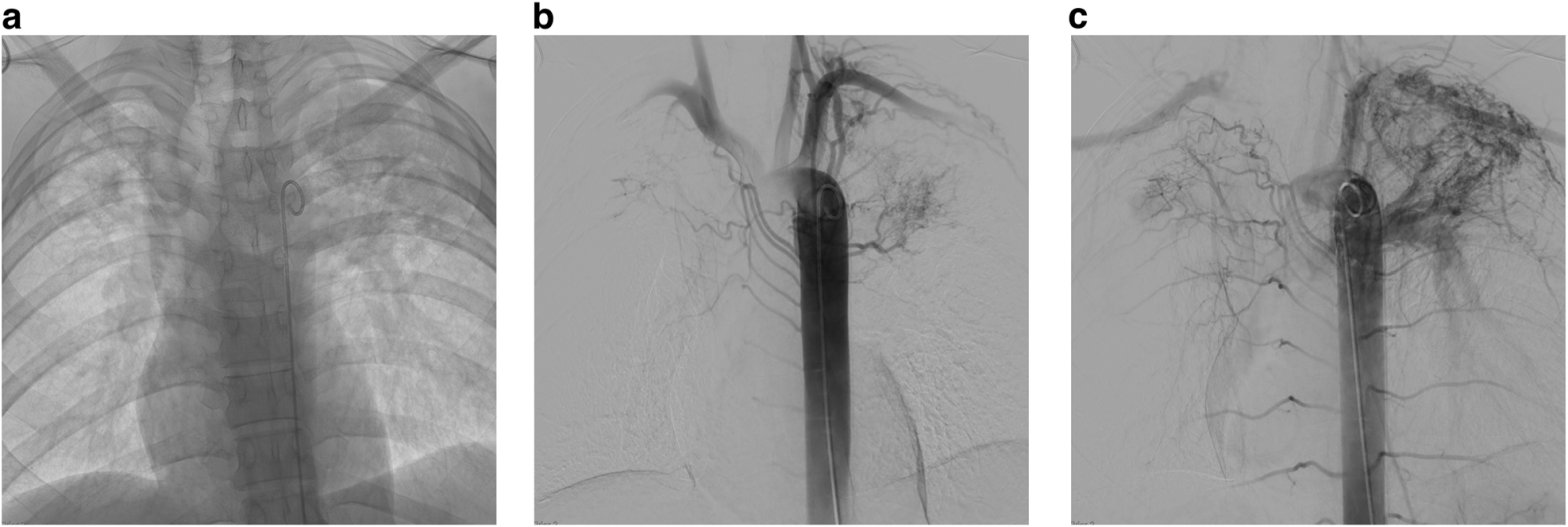
**a** Control image of the thorax during descending thoracic aortography showing bilateral volume loss with increased apical opacity in keeping with mycetomas on a background of old TB. **b**, **c** Digital subtraction arteriogram (DSA) of the aorta demonstrating bilateral bronchial and non-bronchial artery hypertrophy with marked systemic to pulmonary artery shunting. Note the retrograde filling of the left pulmonary artery as well as the right apical pulmonary artery branches

Normal pulmonary parenchyma is supplied by the pulmonary and bronchial arteries, between which there are broncho-pulmonary anastomoses measuring 50–500 µm in diameter [3–6]. In chronic pulmonary inflammatory states, these broncho-pulmonary anastomoses increase in size resulting in pathological bronchial-to-pulmonary artery shunting, with resultant hypertrophy of the bronchial arteries supplying the diseased lung (Fig. 1) [7]. When there is co-existent pleural inflammation, transpleural systemic-to-pulmonary artery communications can form resulting in the hypertrophy of non-bronchial systemic arteries (NBSA), the most common of which are the intercostal arteries, thoracic branches of the subclavian and axillary arteries, and diaphragmatic branches of the inferior phrenic arteries (Fig. 1) [8–10]. Collectively, these systemic arteries expose inflamed fragile peripheral pulmonary arteries to high systemic arterial pressures, increasing the risk of rupture and bleeding [7, 11]. BAE aims to reduce the systemic arterial perfusion pressure to these friable pulmonary vessels, thereby reducing the risk of further hemoptysis [10].

Current opinion advocates that when particles are used for BAE, a particle size of greater than 300 µm should be used to reduce the risk of non-target embolization [12]. It is the opinion of the current authors, however, that the risk of complications due to the use of smaller particles is overstated and that the more distal embolization that is achieved by these agents is likely to be associated with better outcomes in terms of persistent or recurrent hemoptysis. Very few articles report outcomes using either polyvinyl alcohol (PVA) particles or calibrated microspheres smaller than 300 µm [13–16]. The purpose of this retrospective study is to review the outcomes of patients with hemoptysis treated by embolization with 150–250 µm PVA particles.

## Methods

### Study population

A retrospective analysis of all BAE procedures for the treatment of both acute massive and small-volume chronic persistent hemoptysis at a single tertiary center from 2010 to 2022 was performed. This retrospective review of the institution’s practice was approved by the local audit committee under the Health and Social Care Research Framework. All patients aged 16 or over presenting with hemoptysis who underwent BAE (either with or without concurrent pulmonary artery branch embolization) were included. Inclusion criteria for embolization included massive hemoptysis (as defined below) and recurrent less severe bleeding not responding to conservative treatment, particularly if this had increased in frequency or volume. Informed consent was obtained, before all procedures, from all patients. Patient demographic and clinical information were collected from the hospital’s electronic patient records. Imaging and procedural information were obtained from the picture archiving and communication system and radiology information system.

Respiratory function, clotting parameters, and hemodynamic status were optimized (as far as possible) pre-procedure. Patients received tranexamic acid and antimicrobial therapy when appropriate, and anesthetic support was sought where necessary. A pre-procedural aortic-phase contrast-enhanced computed tomography (CT) was performed in most patients to identify the site and etiology of bleeding, evaluate bronchial artery and NBSA hypertrophy, and assess for the presence of concurrent pulmonary artery pseudoaneurysms.

Patients presenting with hemoptysis due to pulmonary artery aneurysmal/pseudo-aneurysmal disease due, for example, to iatrogenic catheter trauma and acute necrotizing pneumonia in whom bronchial artery hypertrophy is not a feature, were not included in our study population.

### Procedural technique

Angiography was conducted via a femoral arterial approach under either local or general anesthesia. A descending thoracic aortogram was performed in most cases followed by selective and super-selective angiography of the abnormal vessels using a 5 Fr Cobra catheter (Cordis) and co-axial microcatheter (2.4 Fr Direxion, 2.0 Fr Truselect, Boston Scientific or 2.7 Fr Progreat, Terumo). Embolization was performed once a stable, non-wedged catheter position was established distal to any important systemic arterial branches supplying adjacent normal vascular beds. PVA particles measuring 150–250 µm (Contour, Boston Scientific) were used as the initial embolic agent in all patients. Larger particles (up to 1000 µm) were subsequently used when appropriate. Post-embolization angiography confirmed successful vessel occlusion and obliteration of systemic-to-pulmonary artery shunting.

When a pulmonary artery branch pseudoaneurysm was present, a 6Fr 90 cm Brite Tip^TM^ sheath (Cordis) was used coaxially with a 5 Fr 100 cm headhunter catheter (Cordis) to catheterize the pulmonary artery via a femoral venous approach. Bronchial or systemic arteriopathy was used to guide cannulation in view of systemic to pulmonary artery shunting. A tri-axial 2.4 Fr Direxion microcatheter (Boston Scientific) was used in some cases and front door/backdoor embolization was performed either using microcoils or Amplatzer vascular plugs delivered through the microcatheter or Headhunter catheter depending on the vessel size. Once embolization was complete, systemic arterial embolization was then performed using PVA particles.

### Definitions

Massive hemoptysis was defined as a total expectorated volume of > 300 mL in 24 h [17]. Technical success was defined as the ability to catheterize and embolize all the visualized hypertrophied bronchial arteries and NBSAs. Clinical success was defined as complete cessation of hemoptysis or a significant reduction in the volume of blood loss, resulting in clinical improvement and no requirement for further embolization or medical intervention within 30 days [12]. Major complications were defined as those requiring unplanned treatment, prolonged hospitalization, or resulting in permanent adverse sequelae or death. Minor complications were self-limiting and required no specific treatment [18].

### Data analysis

Patients were followed up for recurrence and complications until May 2023. For patients who were lost to follow-up or died, the end of the follow-up period was defined as the most recent date for which follow-up information was available or the date of death. All results are expressed as mean or median and interquartile range (IQR). Cumulative survival, hemoptysis-free survival, and hemoptysis recurrence were estimated using the Kaplan–Meier method. A two-tailed unpaired student’s t-test was used to compare continuous variables, while the Chi-square test was used to compare categorical data. *p* < 0.05 was considered statistically significant. Statistical analysis was performed using GraphPad Prism 9.5.1 (San Diego, CA).

## Results

One hundred forty-four patients (86 male and 58 female) with hemoptysis underwent a total of 189 embolization procedures (132 for massive and 57 for small/moderate volume hemoptysis). The mean age was 53 years (range 16–91 years). The spread of etiologies is demonstrated in Table 1.

**Table 1:** Etiologies of hemoptysis with multiple pathologies co-existing in some patients

Etiology	Number of cases
Bronchiectasis	58
Mycetoma	57
Chronic tuberculosis	47
Pulmonary sarcoidosis	11
Pulmonary hypertension	9
Active tuberculosis	7
Malignancy	7
Cystic fibrosis	3
Cryptogenic	6

One hundred seventy-six of 189 procedures were performed under local anesthesia (93%), while 13 were performed under general anesthesia. A descending thoracic aortogram was performed in 153/189 cases (81%). PVA particles were used as the only embolic agent in 171 cases (90%), with 150–250 µm particles used as the sole agent in 137 cases. Adjunctive upsizing to larger particle sizes was performed in 33 cases (up to 355 µm in 11 cases, 500 µm in 18 cases, 710 µm in three cases, and 1000 µm in 1 case). In one case where PVA particles were used as the sole agent, the particle size used was not recorded. A pulmonary artery pseudoaneurysm was present in 17 of 144 patients (11.8%). In 17/189 procedures, concurrent pulmonary artery branch embolization was performed using combinations of coils, NBCA, Amplatzer vascular plugs, and 150–355 µm PVA particles. None of the cases where a pulmonary artery pseudoaneurysm was embolized via a pulmonary arterial approach required repeat embolization.

A total of 424 arteries were treated, of which 209 were bronchial arteries and 215 NBSAs. The median number of arteries treated per patient was 2 [range 0–9] (Table 2). The technical success rate was 97% (185/189). Of the four failed procedures, one case failed due to iatrogenic proximal left bronchial artery dissection that precluded safe distal embolization. One failed due to difficulty catheterizing the left costocervical trunk from a transfemoral approach (embolization was subsequently performed successfully via a brachial approach). An abnormal intercostal artery was not embolized in one case due to the presence of an anterior spinal artery. One procedure was terminated early as the patient was unable to tolerate it to completion.

**Table 2:** Distribution of treated vessels

Artery	Number embolized
Bronchial	209
Intercostobronchial	48
Intercostal	63
Inferior phrenic	23
Internal mammary	31
Other non-bronchial systemic	50

The clinical success rate was 93% (176/189). The median follow-up period was 35 months [IQR 19–89]. Overall, hemoptysis eventually recurred in 54/189 cases, with an estimated overall recurrence rate using the Kaplan–Meir method of 38%. The 30-day, 1-year, 2-year, and 5-year recurrence rates were 7%, 19.6%, 27%, and 34%, respectively. The median time to recurrence was 6 months [IQR 1–19]. Of 57 patients with small-volume hemoptysis, 7 (12%) had ongoing small-volume bleeding episodes. One hundred eighteen patients underwent a single embolization procedure with 26 patients (18%) requiring more than one (17 patients had 2 procedures, 5 had 3 procedures, 1 had 4 procedures, 2 had 5 procedures, and 1 patient had 8 procedures). The median time to repeat intervention was 144 days [IQR 42–441].

The rate of major complications was 1% (2/189). One patient with cystic fibrosis developed a pneumothorax on two occasions after two consecutive embolization procedures under general anesthesia, which both required local hospital intensive care admission. This patient had undergone ten previous embolizations, three of them at another hospital before referral to our center. A pneumothorax occurred after the 9th and 10th embolization procedures. Twenty-two patients (11%) experienced minor complications; 18/189 (9%) suffered self-limiting chest pain post-procedure, one developed a post-embolization syndrome with chest pain, fever, and malaise requiring medical therapy as an outpatient for two weeks, one patient developed a small groin puncture site hematoma, one patient had an iatrogenic left bronchial artery dissection, and another developed a small asymptomatic aseptic splenic infarct. No long-term adverse sequelae were recorded.

**Fig. 2:**
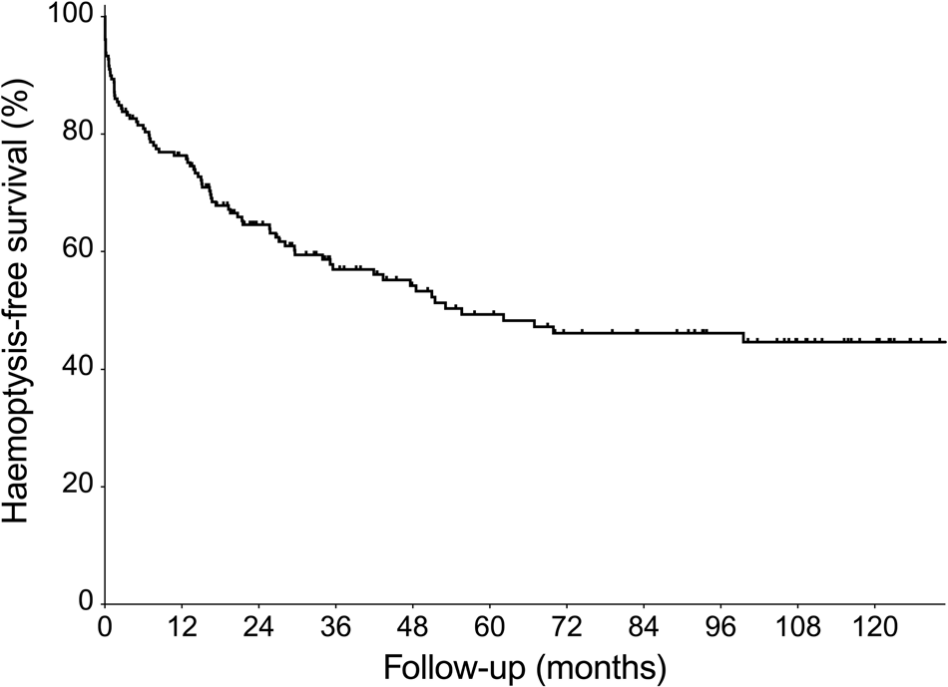
Cumulative hemoptysis-free survival of patients with hemoptysis following BAE (*n* = 189)

**Fig. 3:**
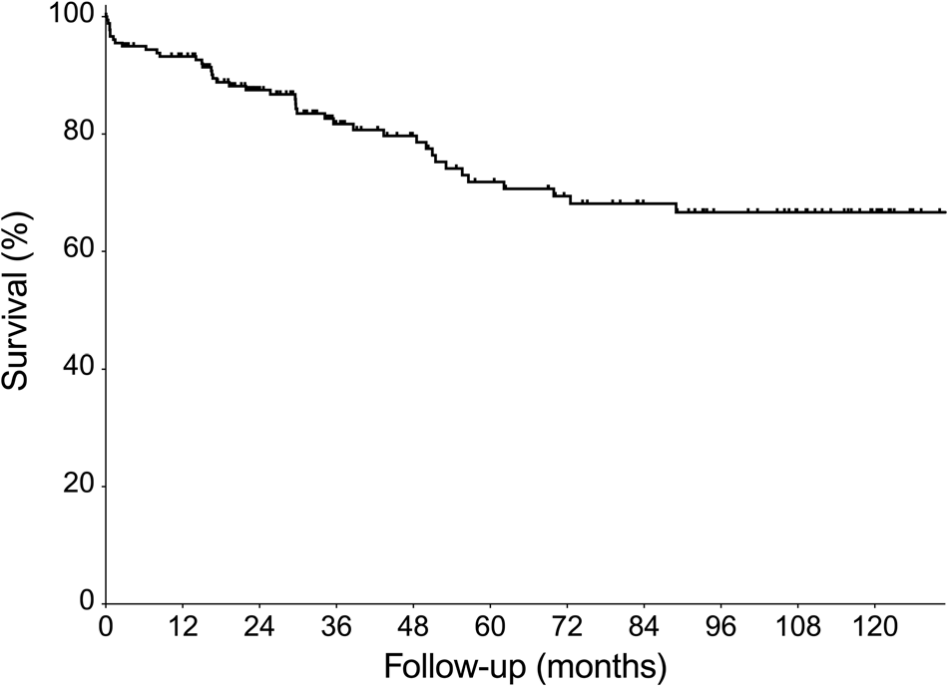
Cumulative survival of patients with hemoptysis following BAE (*n* = 189)

There were 32 deaths during the follow-up period with a median time from embolization to death of 28 months [IQR 8–51]. One hundred twelve (78%) patients were still alive from the initial cohort of 144 patients at the end of the follow-up period (Figs. 2 and 3). Four deaths occurred within 30 days of uneventful procedures that were successful in achieving immediate hemorrhage control (2% 30-day mortality) due to the progression of their underlying disease: cystic fibrosis in one patient, idiopathic pulmonary fibrosis complicated by chronic thromboembolic pulmonary hypertension in another, chronic necrotizing pulmonary aspergillosis on a background of chronic TB in the third, and multiorgan failure on a background of necrotizing pneumonia in the fourth.

The effect of the different variables on 30-day and 1-year recurrence is presented in Table 3. Female patients were less likely to have recurrent hemoptysis at 30 days compared to males 2.4% vs 13.3%, *p* = 0.0317. One-year recurrence was significantly higher in those with massive hemoptysis compared to those with moderate volume/persistent hemoptysis.

**Table 3:** Comparison of 30-day and 1-year hemoptysis recurrence rates with different variables

	Thirty-day recurrence (%)	*p-*value	One-year recurrence (%)	*p*-value
Male (*n* = 106) vs female (*n* = 83)	13.3 vs 2.4	0.0317*	25.3 vs 15.7	0.5761
Massive (*n* = 132) vs moderate/recurrent (*n* = 57)	5.8 vs 1.1	0.2335	13.8 vs 3.7	< 0.0001*
Benign (*n* = 182) vs malignant (*n* = 7)	6.9 vs 0	0.4335	17.5 vs 0	0.1852
Local anesthesia (*n* = 176) vs general anesthesia (*n* = 13)	6.9 vs 0	0.3099	16.4 vs 1.1	0.8381
Pulmonary artery branch pseudoaneurysm (*n* = 17) vs no pseudoaneurysm (*n* = 172)	1.1 vs 5.8	0.404	2.1 vs 15.34	0.4896

*Statistically significant difference

There was no significant difference in patients’ age, number of previous embolizations, or number of lobes involved between those who had recurrent hemoptysis at 30 days and 1 year compared to those without recurrent hemoptysis at the same time points. Having concurrent embolization of a peripheral pulmonary artery pseudoaneurysm was also associated with lower 30-day and 1-year recurrence rates. This was also not statistically significant.

## Discussion

### Bronchial vs pulmonary source of hemorrhage

Great emphasis is placed by some authors on the distinction between a bronchial and pulmonary source of hemorrhage in patients with hemoptysis. It should be recognized, however, that in patients with chronic inflammatory lung disease, bleeding originates from friable diseased pulmonary arteries in areas of chronic lung inflammation that are exposed to systemic arterial pressure from hypertrophied bronchial and non-bronchial systemic arteries and should, therefore be recognized as ‘pulmonary bleeding’. At a microscopic level, there are probably many pseudoaneurysms of tiny peripheral pulmonary artery branches; when the lung disease is particularly severe, however, larger pseudoaneurysms develop which become visible on angiography and occasionally on CT. The patients in this series with visible pseudoaneurysms are, therefore, at the more severe end of a spectrum of the same underlying disease process as all the other patients included in this paper.

### Rationale for the use of small particulate PVA for BAE

A variety of embolic agents are used in BAE including gelfoam, coils, PVA particles, calibrated microspheres, and N-butyl cyanoacrylate (NBCA). PVA particles are the most commonly used agent [12] and are effective in the short- to medium-term, although recanalization is common [6, 12, 19]. The recommendation that a particle size of greater than 300 µm should be used for BAE is due to the feared risk of non-target embolization, which is discussed in detail below. The disadvantage of using larger particles, however, is that these will cause a more proximal embolization of hypertrophied bronchial and NBSA than is ideal, as their more distal branches are likely to remain perfused by small systemic artery branches that are not amenable to embolization resulting in higher rates of recurrent bleeding [12, 20, 21].

Small particles will achieve a more distal embolization closer to the site of the friable pulmonary artery branches from which bleeding occurs and distal to other systemic artery anastomoses. This will reduce the perfusion pressure to areas of inflamed lung to a greater extent than would be achieved with a more proximal embolization. In addition, recanalization (and therefore bleeding recurrence) is less likely when small vessel thrombosis occurs due to distal luminal occlusion than embolization of a more proximal supplying vessel.

### The risk of non-target embolization with small particles is probably overstated

Non-target embolisation during BAE can occur via several routes and it is worthwhile looking at these in turn:

- Through hypertrophied bronchopulmonary anastomoses into peripheral pulmonary artery branches with the feared risk of pulmonary infarction [6, 7, 11, 21, 22].

Normal bronchopulmonary anastomoses measure between 50 µm and 500 µm and whilst these are likely to increase in size in inflammatory disease states, most remain small and are successfully occluded with 150–250 µm PVA. It is highly likely, however, that some particles will pass through larger anastomoses into pulmonary artery branches, and it is important that frequent diagnostic arteriograms are performed at intervals during the embolization of bronchial and NBSAs to assess progress. If there is persistent bronchopulmonary artery shunting after commencing embolization with small particle PVA, larger particles can then be used as was required in 33 of 189 procedures in this series. The relatively small number of PVA particles that will have passed into pulmonary artery branches in the area of diseased lung are very unlikely to have any long-term effect as confirmed in this series where there were no instances of symptomatic pulmonary infarction.

**Fig. 4:**
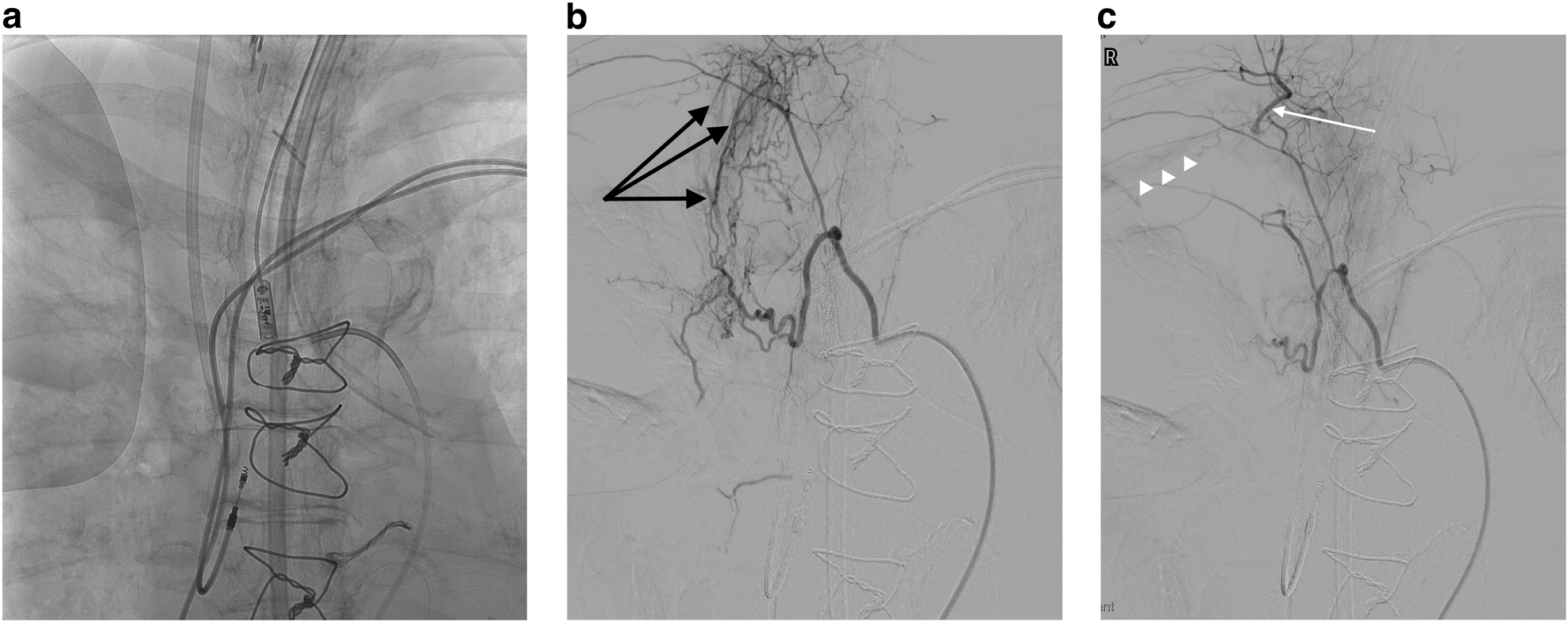
**a** Control image of the right upper chest. **b** DSA of a mildly hypertrophied bronchial branch of the right intercostobronchial (ICB) artery trunk in a patient with presumed vasculitis and massive hemoptysis demonstrating bronchial to pulmonary artery (black arrows) shunting. **c** Completion arteriography after embolization has been performed of the right bronchial artery using a co-axial catheter in a free-flow position, demonstrating obliteration of shunting. Note that the catheter is in a wedged position within the proximal ICB trunk resulting in retrograde filling of the costocervical trunk (white arrow) back to its origin with faint opacification of the right subclavian artery (white arrowheads)

- Via normal systemic arterial branches supplying, for example, the spinal cord, the bronchi, the esophagus, and the vasa vasorum of the aorta and pulmonary arteries, which may arise from the bronchial arteries that are being embolized [7, 12, 23];
- and via normal anastomoses with adjacent systemic arterial beds such as those that occur between intercostal branches of the right intercostobronchial trunk and intercostal branches of the costocervical trunk (Fig. 4), where there is a risk of upper limb ischemia and posterior circulation cerebrovascular events due to proximity of the vertebral artery [24, 25].

Serious complications due to non-target embolization of these normal arterial branches and anastomoses are almost invariably technique-related due to the injection of embolic material through a catheter in a wedged position, which forces the emboli into important small vessels that would not be occluded if embolization is performed with the catheter in a ‘free-flow’ position. Great care must be taken, as it was in all the embolization procedures in this series, to avoid embolization being performed from a wedged catheter position, and this is likely the reason why there were no cases of spinal cord ischemia, stroke, or non-target embolization of other vascular territories causing symptoms in this study.

Complication rates observed in this study (11% minor and 1% major) are comparable with pooled complication rates for BAE presented in a recent systematic review and meta-analysis (10% and 2% for minor and major complications, respectively) [26]. The most commonly reported complications include chest pain, back pain, dysphagia, and post-embolisation syndrome consisting of fever and leukocytosis, all of which are typically self-limiting [16]. Of the minor complications observed here, the majority consisted of transient mild chest pain, which occurred almost exclusively in those patients in whom intercostal and inferior phrenic artery embolization was required. Rare literature reports of major complications can all be attributed to non-target embolization and include bronchial infarction, esophago-bronchial fistula, myocardial ischemia, ischemic colitis, stroke, and spinal cord ischemia [12], with the last of these reported to occur in between 0.6-4.4% cases in a recent systematic review [16]. None of these complications were observed in the present study.

This study establishes that BAE performed using small PVA particles of 150–250 µm is safe and effective, achieving high rates of technical and clinical success, and low rates of recurrent hemoptysis and complications. Prior studies that describe the use of larger (> 300 µm) PVA particles as the principal embolic agent report 76–100% technical success and 64–94% clinical success [13–15, 20]. This compares with the technical and clinical success rates of 97% and 93% respectively in the present study. Of the most recent large studies with long-term follow-up, Frood et al reported technical and clinical success rates of 90% and 86.5% respectively for 68 patients undergoing 96 BAE procedures between 2000 and 2012 using 300–500 µm PVA particles [27]. Dorji et al reported technical and clinical success rates of 92.4% and 70.1% respectively for 145 patients undergoing 184 procedures from 2008 to 2018 using 355–500 µm PVA particles [28].

Whilst BAE does not address the underlying disease process, long-term hemoptysis control can be achieved with a combination of optimal medical therapy and repeat BAE. Early hemoptysis recurrence is typically secondary to incomplete embolization due to a failure to identify and treat all hypertrophied bronchial and NBSAs and focal pulmonary arterial pseudoaneurysms [29]. Late recurrence is generally due to the recanalization of previously embolized vessels and/or the development of new bronchial arteries/NBSAs due to the progression of the underlying pulmonary disease or the permanent proximal occlusion of previously embolized systemic arteries.

The 30-day and overall hemoptysis recurrence rates of 7% and 38%% achieved in the present study using small 150–250 µm PVA particles are low in comparison with published data using larger particles (reported figures range from 10% to 30% and 10 to 60% for 30-day and overall recurrence, respectively [11, 26, 30, 31]). They are comparable with some of the lowest early recurrence rates reported by Agmy et al (10% 30-day recurrence using 355–500 µm PVA particles and coils [13]) and Bhalla et al (7% recurrence at 2 months using 300–1000 µm PVA particles, occasionally supplemented with gelfoam and NBCA [14]).

Embolization with NBCA is associated with some of the lowest reported recurrence rates in the literature [20, 32, 33]. A comparative study by Woo et al reported a recurrence rate at 5 years of 17% for NBCA vs 34% for PVA [20], and Yoo et al reported a recurrence rate of 8% at one month, 17% at 1 year, 19% at 2 years, and 43.8% at 5 years using NBCA as a sole agent [33]. The overall recurrence rates achieved here using 150–250 µm PVA particles are comparable to some of the studies using NBCA and are favorable, particularly given the fact that NBCA is considerably more difficult to use.

**Fig. 5:**
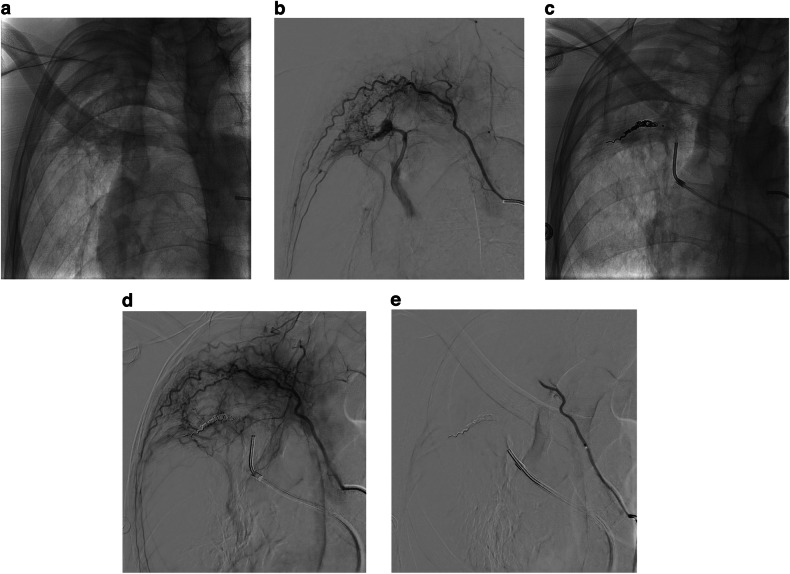
**a** Right anterior oblique control image of the right upper chest showing right apical scarring and a mycetoma. **b** DSA of a right upper intercostal artery demonstrating marked systemic to pulmonary artery shunting and a pulmonary artery branch pseudoaneurysm. **c** and **d** Control image, followed by an intercostal arteriogram following coil embolization from the pulmonary arterial side demonstrating occlusion of the pulmonary artery. **e** Completion DSA following embolization of the intercostal artery with 150–250 µm PVA particles obliterating systemic to pulmonary artery shunting

The other point worthy of discussion raised by this study is the incidence of a pulmonary arterial source of hemoptysis requiring embolization via a pulmonary arterial approach. A pulmonary artery pseudoaneurysm was present in 17 of 144 patients (11.8%), several of which were only seen due to the presence of significant bronchial-to-pulmonary artery shunting on selective bronchial or systemic non-bronchial angiograms (Fig. 5). This reinforces the finding of a previous study [29] that such pathology occurs in a significant number of patients with hemoptysis referred for embolization and should be actively sought.

Limitations of this study include its retrospective, observational, and single-center nature. The patient population was inhomogeneous in terms of disease etiology and severity. Management of the underlying pathology mostly occurred at local institutions therefore detailed information about disease status and treatment was not available for all. Severe disease and suboptimal treatment would contribute to higher recurrence rates. Additionally, complete long-term follow-up was also not available for all patients. However, as a tertiary referral center, it is likely that subsequent presentations with significant hemoptysis to a local institution would have been re-referred to our center, therefore significant recurrences are unlikely to have been missed.

## Conclusion

This study demonstrates the safety and efficacy of BAE using 150–250 µm PVA particles with high technical and clinical success rates, good long-term outcomes, and low 30-day and overall hemoptysis recurrence rates. The risk of non-target embolization with small particles is probably overstated so long as a meticulous embolization technique is used avoiding a wedged catheter position and performing frequent diagnostic arteriograms during embolization of individual vessels to assess the progress of embolization.

## Abbreviations

BAE: Bronchial artery embolization
CT: Computed tomography
IQR: Interquartile range
NBSA: Non-bronchial systemic arteries
PVA: Polyvinyl alcohol
